# Creating nanoscale emulsions using condensation

**DOI:** 10.1038/s41467-017-01420-8

**Published:** 2017-11-08

**Authors:** Ingrid F. Guha, Sushant Anand, Kripa K. Varanasi

**Affiliations:** 1 Department of Electrical Engineering and Computer Science, Massachusetts Institute of Technology, Cambridge, MA 02139 USA; 2 Department of Mechanical & Industrial Engineering, University of Illinois at Chicago, Chicago, IL 60607 USA; 3 Department of Mechanical Engineering, Massachusetts Institute of Technology, Cambridge, MA 02139 USA

## Abstract

Nanoscale emulsions are essential components in numerous products, ranging from processed foods to novel drug delivery systems. Existing emulsification methods rely either on the breakup of larger droplets or solvent exchange/inversion. Here we report a simple, scalable method of creating nanoscale water-in-oil emulsions by condensing water vapor onto a subcooled oil-surfactant solution. Our technique enables a bottom-up approach to forming small-scale emulsions. Nanoscale water droplets nucleate at the oil/air interface and spontaneously disperse within the oil, due to the spreading dynamics of oil on water. Oil-soluble surfactants stabilize the resulting emulsions. We find that the oil-surfactant concentration controls the spreading behavior of oil on water, as well as the peak size, polydispersity, and stability of the resulting emulsions. Using condensation, we form emulsions with peak radii around 100 nm and polydispersities around 10%. This emulsion formation technique may open different routes to creating emulsions, colloidal systems, and emulsion-based materials.

## Abstract (web summary)

Emulsions—stabilized mixtures of immiscible liquids—are found in many products, ranging from pharmaceuticals to food. Here Guha et al. propose a simple emulsification method where water vapor is condensed onto oil with surfactant, producing a water-in-oil emulsion with droplets as small as 100 nm.

## Introduction

Emulsions^1, 2^—the dispersion of one liquid phase within a second immiscible host liquid—appear in numerous applications, including a range of drug delivery systems^1, 3–9^, cosmetics^10, 11^, processed foods^12–15^, fuels^16^, and materials fabrication^17^. Key emulsion properties (rheology, stability, transparency, and so on) are intimately linked with the sizes of their dispersions. For example, nanoscale emulsions^2, 18^ consisting of dispersed nanometric droplets (<500 nm in diameter) remain emulsified longer than larger-scale dispersions because they are not prone to gravity separation or other body forces over time^19–21^. Thus, products made with nanoscale emulsions tend to have much longer shelf lives than products with larger-size dispersions.

Emulsification techniques may be broadly classified into two categories: high-energy techniques and low-energy techniques. The most common high-energy emulsification technique is sonication^22, 23^, which typically subjects the emulsion contents to high shear stress during emulsification. Low-energy techniques include phase inversion^24, 25^, flow focusing^26, 27^, liquid-liquid nucleation^28, 29^ (also known as the Ouzo effect), and newly demonstrated approaches, such as bubble bursting^30^. Low-energy emulsification techniques often constrain the scope of materials that may be used for emulsification. For example, the phase inversion technique requires specific solubility requirements of the emulsion components, such that thermal cycling may be used to trigger phase separation. Hence, new emulsification approaches are needed to broaden the range of possible materials formulations and operating conditions.

In this study, we present a bottom-up assembly approach to creating nanoscale emulsions that spontaneously separates condensed nanometric water droplets with thin films of oil as they nucleate. To create this bottom-up assembly, we condense nanoscale water droplets onto surfactant-oil mixtures. While vapor condensation on pure oils has been investigated before^31–34^, we show that condensation on surfactant-rich oil can lead to the formation of highly monodisperse nanoscale emulsions. This method of nanoscale emulsion formation is conceptually simple, scalable, and applicable to a wide range of liquids. We show that the emulsion size and polydispersity may be controlled through the surfactant concentration. Nanoscale emulsions formed via condensation remain dispersed for months, though dynamic light scattering (DLS) measurements performed after several months reveal that the peak radius and polydispersity may shift slightly over time.

## Results

### Emulsion formation via vapor condensation

**Fig. 1:**
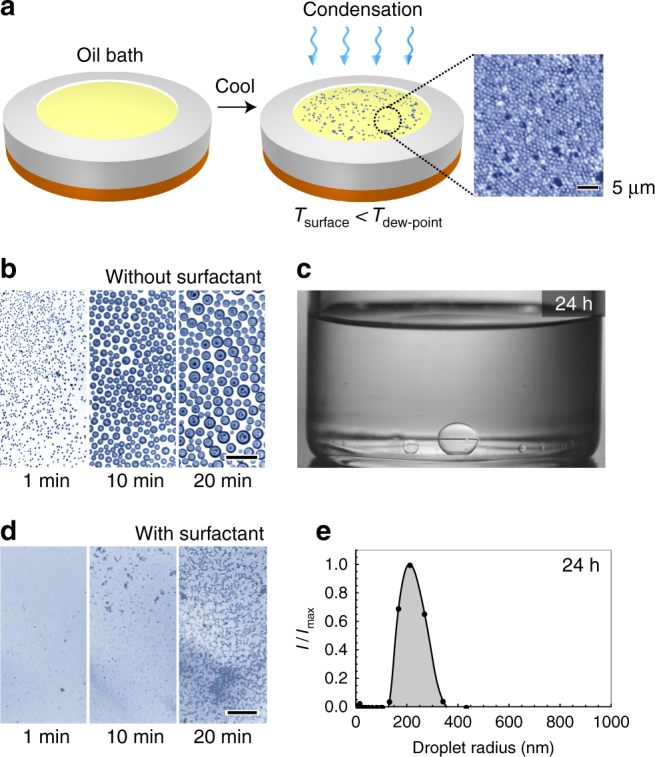
Formation of nanoscale emulsions via condensation. **a** Experimental set-up: an oil bath is placed in a high humidity, temperature-controlled environment and then cooled below the dew point. Water droplets spontaneously condense onto the surface of the oil from the vapor phase. Optical image shows water-in-oil emulsions formed after 10 min of condensation (oil phase: dodecane with 0.1 mM Span 80). Scale bar depicts 5 μm. **b** In the absence of surfactant, water droplets continually coalesce and grow on the surface of dodecane. **c** Without surfactant, the water phase eventually pools as one large drop at the bottom of the dodecane reservoir. **d** When surfactants are added to dodecane, a stable water-in-oil emulsion results after 30 min of condensation (oil phase: dodecane with 100 mM Span 80). **e** DLS measurement taken 24 h after the condensation process with surfactant shows the resulting emulsion has a peak radius of 215 nm and a polydispersity of 20% (oil phase: dodecane with 100 mM Span 80, condensation time: 30 min). Scale bars in parts **b** and **d** depict 50 μm

In our experiments, we place an oil bath on a peltier cooler in a high-humidity chamber (relative humidity 75–80%) maintained at 20 °C. We use dodecane as the model oil in our studies because of its low vapor pressure and low water solubility. For the stabilizing agent, we use Span 80 (sorbitan monooleate), a non-ionic oil-soluble surfactant commonly used in water/oil emulsion studies^35^. To initiate condensation on the oil-surfactant solution, the peltier cooler temperature is decreased to 2 °C. When the temperature of the oil drops below the dew point (13 ± 1 °C), water droplets spontaneously condense on the oil/air interface via heterogeneous nucleation (Fig. 1a). A water-in-oil emulsion results.

We find that the presence of surfactant in the oil is critical to the formation of stable emulsions. When water vapor condenses on pure dodecane, the condensed water droplets continually coalesce and grow indefinitely. The longer the time of condensation, the larger the water drops become (Fig. 1b). Regardless of the total time of condensation, the resulting dispersion is quite unstable; the water separates completely from pure dodecane and forms a few large water drops (Fig. 1c, Supplementary Movie 1). However, if the oil bath contains a sufficient amount of surfactant, the resulting product changes significantly. Figure 1d shows optical images of water condensed onto dodecane containing 100 mM Span 80. In contrast to condensation on pure dodecane, individual water droplets in this case cannot be resolved using optical microscopy. Rather, the oil surface becomes cloudy (Supplementary Movie 1). After 30 min of continuous condensation, the solution was collected and stored in ambient room conditions for 24 h. DLS measurements of this solution revealed the presence of nanoscale water droplets with a peak radius of 215 nm and a polydispersity of approximately 20%, indicating that the solution is a water-in-oil nanoscale emulsion (Fig. 1e).

### Mechanism of emulsion formation

To form stable water-in-oil emulsions by condensing water onto oil, some surfactant must be present in the oil phase. In this emulsification process, the surfactant serves two purposes: to stabilize neighboring droplets once they are formed, and to cause the oil phase to spread across the water/air interface and encapsulate condensed water drops. Both properties are interrelated and dependent on surfactant concentration; however, we address each point separately to emphasize the crucial role of the surfactant in this condensation emulsification process.

This first point describes the general use of surfactants in emulsification; the surfactant stabilizes the emulsion by adsorbing at the oil/water interfaces and decreasing the interfacial tension, creating a surfactant-rich lipid bilayer that increases the energetic barrier to coalescence between neighboring droplets^19, 36^. Thus, the surfactant inhibits coalescence of droplets within an emulsion. In general, if the surfactant concentration exceeds the critical micelle concentration (CMC), the emulsion remains stable^19^. This role of the surfactant is not unique to this emulsification method but rather describes the general role of surfactants in all emulsion systems.

However, the second role of the surfactant is particular to this emulsification method. When an oil is subcooled below the dew point in a humid environment, nucleation of water droplets occurs at the oil/air interface^34^. The subsequent droplet growth process is strongly dependent on the spreading behavior of oil across the water/air interface^34, 37^. The spreading behavior of oil on water can be understood through the spreading coefficient, which is given by *S*_ow_ = *γ*_wa_-*γ*_ow_-*γ*_oa_, where *γ*_wa_, *γ*_ow_, and *γ*_oa_ are the water/air, oil/water, and oil/air interfacial tensions, respectively^34, 37^. In this system, the spreading coefficient predicts whether the oil phase will spontaneously spread over the surfaces of condensed water drops at the water/air interface, cloaking the water drops with a thin film of oil. A positive spreading coefficient indicates the oil will spread; a negative spreading coefficient predicts the oil will not spread^34^.

Since surfactants affect the oil/water interfacial tension, their presence impacts the spreading coefficient. When Span 80 is added to dodecane, *γ*_ow_ drops significantly, decreasing from around 50 mN m^−1^ (no surfactant) to 3 mN m^−1^ (100 mM Span 80). However, *γ*_oa_ and *γ*_wa_ remain roughly constant at 25 mN m^−1^ and 70 mN m^−1^, respectively, as Span 80 does not lower the oil/air interfacial tension or partition at the water/air interface. Therefore, as the surfactant concentration increases, *γ*_ow_ decreases, thereby increasing the spreading coefficient.

**Fig. 2:**
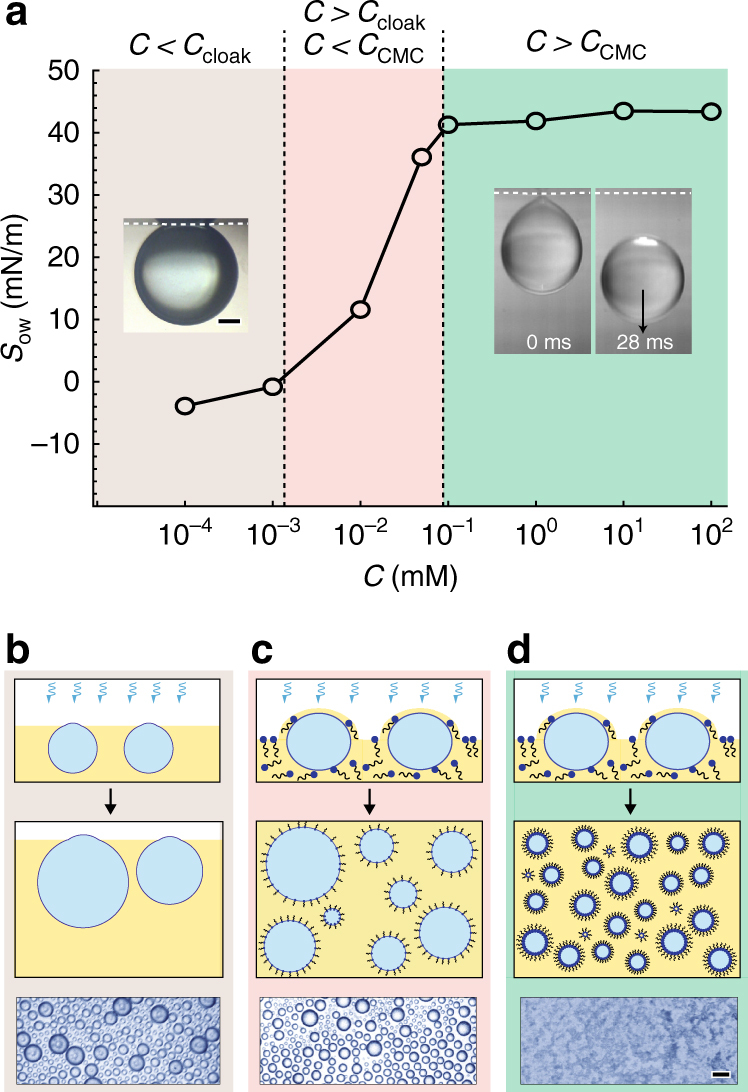
Mechanism of emulsion formation via cloaking. **a** Plot of spreading coefficient as a function of surfactant concentration (*C*) in oil. At low concentrations (brown region: *C* < *C*_cloak_), oil does not cloak water. Inset picture in this regime shows a water droplet balanced at a dodecane/air interface, suspended from the surface tension forces in a non-cloaked state. At high concentrations (green region: *C* > *C*_CMC_), oil spreads over water and the surfactant stabilizes the emulsions. Inset picture in this regime shows a water drop falling from an interface where the oil phase (dodecane with 1 mM Span 80) cloaks the water. At intermediate concentrations (pink region: *C*_cloak_ < *C* < *C*_CMC_), the oil phase cloaks water drops but the surfactant inadequately stabilizes emulsions, resulting in large polydisperse emulsions. Scale bar shows 500 μm. **b**–**d** Schematics and microscopic images of the three corresponding regimes presented in part **a**. Part **b** shows low surfactant concentrations (*C* < *C*_cloak_), part **c** shows intermediate surfactant concentrations (*C*_cloak_ < *C* < *C*_CMC_), and part **d** shows high surfactant concentrations (*C* > *C*_CMC_). Optical images show emulsions formed after 5 min using 10^−4^ mM Span 80 (part **b**), 10^−2^ mM Span 80 (part **c**), and 1 mM Span 80 (part **d**) in dodecane. Scale bar represents 50 μm

Figure 2a shows a plot of the spreading coefficient *S*_ow_ as a function of the Span 80 concentration in dodecane. *S*_ow_ transitions from negative to positive around a concentration of 10^−3^ mM. At this critical concentration, the oil phase spontaneously spreads over water, effectively cloaking the water droplet. We hereafter refer to this critical surfactant concentration as *C*_cloak_. Below this concentration, a small water droplet gently placed at the oil/air interface pins at the surface, as the interfacial tension forces dominate over the effects of gravity (Fig. 2a, inset image in low surfactant concentration regime). Above this concentration, a water droplet gently placed at the oil/air interface soon becomes cloaked by the oil-surfactant solution and spontaneously submerges (Fig. 2a, inset images in high surfactant concentration regime). In the case of nucleated droplets, the spreading time is on the order of 10^−12^ to 10^−9^ s, demonstrating that water droplets are quickly encapsulated by the oil and submerged within the oil medium^34^. This spontaneous cloaking enables a bottom-up assembly approach to forming nanoscale emulsions by enabling the submerging and dispersing of water drops within the oil phase.

As illustrated in Fig. 2b–d, the concentration of surfactant in the oil may fall into one of three regimes: below *C*_cloak_ (Fig. 2b), between *C*_cloak_ and the concentration of CMC (*C*_CMC_, which occurs around 0.1 mM) (Fig. 2c), and above *C*_CMC_ (Fig. 2d). Below *C*_cloak_, unstable emulsions result from condensation (Fig. 2b, optical image). The water droplets continually grow during the condensation process and do not emulsify with the oil phase. Between *C*_cloak_ and *C*_CMC_, unstable polydisperse microscale emulsions result from condensation (Fig. 2c, optical image). In this regime, the oil phase spontaneously spreads over the surfaces of nucleated water droplets, cloaking them with a thin film of oil. The water droplets become spontaneously dispersed within the oil medium, though the surfactant concentration is insufficient to stabilize the droplet size. As the surfactant concentration increases above *C*_CMC_, the emulsions become increasingly monodisperse and stable and eventually remain nanometric at sufficiently high surfactant concentrations (1 mM or higher). In this range, the condensed droplets create a hazy, indistinct swirling pattern on the surface of the oil (Fig. 2d, optical image).

### The role of surfactant concentration and condensation time

**Fig. 3:**
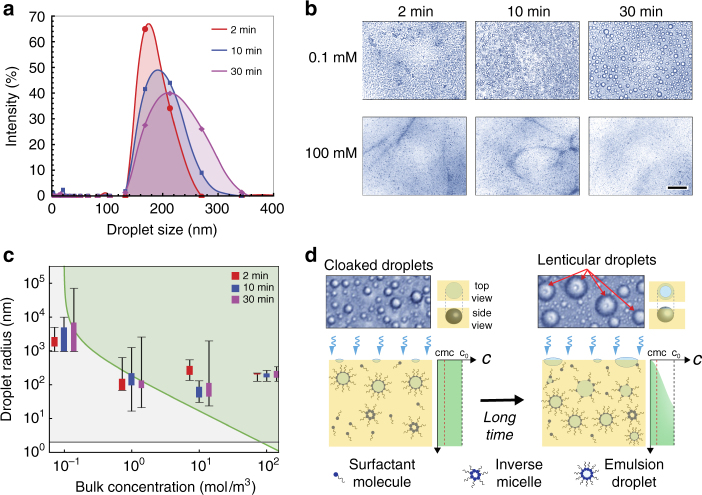
Emulsion size dependence on surfactant concentration and condensation time. **a** Droplet size distributions formed by condensation of water on dodecane with 100 mM Span 80. As the condensation time increases, the peak radius increases slightly and the size distribution broadens. **b** Optical images of emulsions at various condensation times formed using different surfactant concentrations in oil. With low surfactant concentrations (0.1 mM), the water drops visibly grow to several micrometers in diameter. With high surfactant concentrations (100 mM), the water drops remain nanometric at the same condensation times. **c** Droplet size distributions for emulsions formed using different surfactant concentrations over various condensation times. Colored bars span the peak radius ± one standard deviation. End caps indicate the minimum and maximum droplet size detected. Size distributions are overlaid with theoretical prediction of emulsion radius vs. surfactant concentration (plotted as a green line). The green region shows where the emulsion is theoretically stable, whereas the light grey region shows where the emulsion is theoretically unstable, due to insufficient surfactant. The raw DLS measurements are shown in supporting information (Supplementary Figs. 1–3). **d** Effect of free surfactant depletion during condensation. At small timescales, condensed droplets are completely cloaked with oil. At large timescales, the droplets become uncloaked as surfactant depletes in the region close to the oil/air interface. The droplets adopt a lenticular shape

To further understand this emulsification method, we systematically vary two main experimental parameters when forming nanoscale emulsions using condensation: the concentration of Span 80 in dodecane and the time of the condensation process. Figure 3a shows the DLS measurements of nanoscale emulsion size distributions resulting from 2, 10, and 30 min of condensation on 100 mM Span 80 in dodecane. As the time of condensation increases, the peak radius of the emulsion increases slightly, and the size distribution broadens. Figure 3b shows top-down optical images of the emulsion formation using a low surfactant concentration (0.1 mM) and a high surfactant concentration (100 mM) at different times of condensation. The resulting emulsions are much larger for the 0.1 mM case. The individual water emulsions are several micrometers large, whereas individual emulsions cannot be resolved optically in the 100 mM case. Figure 3c shows the DLS measurements of size distribution for nanoscale emulsions formed using various concentrations of Span 80 and times of condensation. Experimentally we observe that the amount of surfactant in the oil must be quite high (at least 1 mM, or in other words at least 10 × *C*_CMC_) to produce stable nanoscale emulsions.

Interestingly, the cloaking behavior of the oil changed over long periods of condensation times (>30 min). Figure 3d contains optical images taken during the condensation process. Here the oil composition is 0.1 mM Span 80 in dodecane (100 × *C*_cloak_). The image on the left shows condensed water drops after two minutes of condensation; the water drops are visibly cloaked with oil. The image on the right shows the condensed water drops after 100 min of condensation. Here the water drops visibly become uncloaked. In the center region on top of the water drops, lenses appear. These areas show where the water/air interface has been restored.

### Theory of surfactant concentration and emulsion size

To estimate the minimum concentration of surfactant required to stabilize a particular emulsion size, we consider a simple mass balance of surfactant before and after condensation:

1 $$N_{\mathrm{A}}V_{{\mathrm{oil}}}C_{\mathrm{b}} = \Gamma _{\mathrm{s}}A_{{\mathrm{w/o}}} + N_{\mathrm{A}}V_{{\mathrm{oil}}}C_{{\mathrm{CMC}}},$$

where *V*_oil_ is the volume of oil in the emulsion, *C*_b_ is the bulk surfactant concentration in the oil, *Γ*_s_ is the surfactant packing density at the water/oil interface, *A*_w/o_ is the water/oil interfacial area within the emulsion, and *N*_A_ is Avogadro’s number. Before condensation, the total amount of surfactant in the system can be calculated by multiplying the volume of the oil *V*_oil_ by the bulk surfactant concentration *C*_b_. After condensation, at equilibrium some of the surfactant is adsorbed at the water/oil interfaces and some remains in the oil. The first term describes the amount of surfactant adsorbed at the interfaces: the surfactant packing density at the interface *Γ*_s_ multiplied by the water/oil interfacial area *A*_w/o_. The second term describes the minimum amount of surfactant remaining in the oil phase necessary for emulsion stability: the volume of oil *V*_oil_ multiplied by the amount of free surfactants in solution. If the initial surfactant concentration is below the critical micelle concentration, then the free surfactant concentration after condensation is approximated as the initial surfactant concentration, *C*_b_. If the initial surfactant concentration exceeds the critical micelle concentration, then the free surfactant concentration after condensation is approximated as the critical micelle concentration, *C*_CMC_.

If we let the ratio of water volume *V*_w_ to oil volume *V*_oil_ in the emulsion be *k = V*_w_ */V*_oil_, and if we approximate that the emulsion consists entirely of monodisperse spheres, then we may calculate the minimum droplet radius that can be supported by a given initial concentration of surfactant in the oil bulk:

2 $$r_{\mathrm{w}} = 3k\frac{{\Gamma _{\mathrm{s}}}}{{C_{\mathrm{b}} - C_{{\mathrm{CMC}}}}}.$$

As expected, as the bulk concentration of surfactant increases, the minimum predicted sustainable emulsion size decreases. Figure 3c plots this minimum emulsion radius as a function of surfactant concentration. The green region (including this curve) indicates the region where emulsions are stable (where sufficient surfactant is present to stabilize the oil-water interfacial area). In this plot, we use an experimentally obtained packing density for this oil/surfactant system: *Γ*_s_=5 × 10^16^ molecules per m^2^ ^38^. The value of *k* was obtained through careful mass measurement experiments, where we found a water condensation rate of 0.011 mL min^−1^ using our experimental conditions. Using a constant oil volume (*V*_oil_ = 8 ml), we found *k* = 0.1 for a condensation time of approximately 10 min.

Depending upon supersaturation conditions, the critical nuclei during condensation are typically around 2–10 nm in size. Therefore, this size range represents the theoretical lower size limit of water droplets condensed onto the oil surface that may be dispersed within the emulsion. However, it is interesting to note that the minimum peak radius we measured was around 100 nm. Although the oil/water droplet interfaces are densely packed with Span 80, the surfactant shell only accounts for a few nanometers of the measured radius given the length scale of the surfactant. For low surfactant concentrations, the surfactant mass conservation prediction follows the experimental data closely. However, at high surfactant concentrations, the prediction deviates significantly from the experimental outcome. The emulsion radius plateaus around 100 nm; additional surfactant does not decrease the measured emulsion size (Fig. 3b). We hypothesize that this deviation between measurements and the model is most likely explained by the dynamics of surfactant adsorption at the water/oil interfaces^39, 40^. As water condenses, surfactant molecules continually adsorb at the oil/water interfaces of newly formed droplets. This adsorption creates a local depletion of free surfactants in the oil near the oil/air interface, precisely where condensation occurs. Therefore, the local free surfactant concentration near the oil/air interface during condensation may be significantly lower than the initial bulk surfactant concentration in the oil, and this surfactant depletion may limit the minimum emulsion size that can be stabilized under these conditions using our experimental setup. In practical systems, these limitations of surfactant depletion may be overcome by infusing surfactant near the oil/air interface, or by an alternative scalable approach to maintaining the bulk surfactant concentration close to the region of condensation. Further investigation is needed to understand the precise dynamics of surfactant depletion and its role in limiting the minimum producible emulsion size.

Our observation of lenticular water drops after long periods of condensation provides further evidence of surfactant depletion (Fig. 3d). Pure dodecane does not spread on water. However, when the surfactant concentration exceeds 10^−3^ mM, the oil-surfactant mixture spreads on the water droplets (Fig. 2a). A water droplet that nucleates and grows on a non-spreading oil has a distinct lenticular shape exhibiting two concentric circles (Figs. 1b, 2b). By contrast, a water droplet that nucleates and grows on a spreading oil has a distinct spherical shape (Fig. 2c). Thus the shape of the droplet is directly related to the oil/water interfacial tension and the local surfactant concentration around the droplet. In an experiment where we condense water droplets onto an oil-surfactant mixture containing 0.1 mM Span 80 (100 × *C*_cloak_), we observe that the initial water droplets have a completely spherical appearance. However, with time the spreading coefficient appears to transition from positive to negative during long condensation processes, as the oil phase recedes from the tops of the water droplets and the droplets transition into a lenticular shape. This behavior indicates that the local surfactant concentration around the droplet near the oil/air interface depletes to less than *C*_cloak_ (10^−3^ mM) during the course of the experiment. These results illustrate the complex interplay between formation of nuclei at the oil/air interface, surfactant diffusion within the bulk, and surfactant adsorption at the water/oil interface. Notably, the surfactant depletion appears only as a localized effect during emulsion formation near the oil/air interface after long condensation times. Once the droplets have formed and dispersed within the oil phase, the presence of bulk surfactant at concentrations above *C*_CMC_ sufficiently stabilizes the emulsion size.

**Fig. 4:**
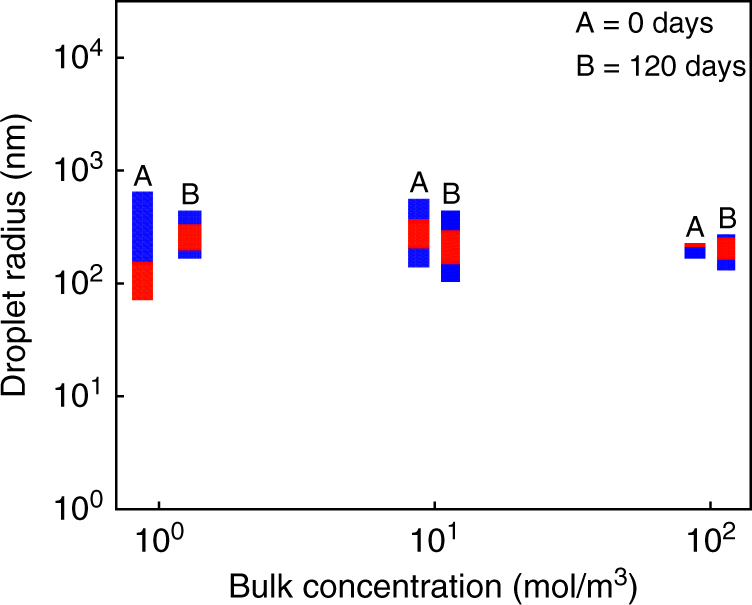
Emulsion stability over a period of 120 days. Long-term measurements of peak radii and polydispersities of nanoscale emulsions formed using water condensation on different concentrations of Span 80 in dodecane for condensation time of 2 min. Bars marked A show DLS data taken the same day the emulsion was made, or 0 days after the emulsion was formed; bars marked B show DLS data taken 120 days after the emulsion was formed. Red region: peak droplet radius ± one standard deviation; blue region: range of minimum to maximum droplet size detected. The raw DLS measurements for droplet sizes corresponding to 120 days is shown in supporting information (Supplementary Fig. 4)

To investigate the long-term stability of these emulsions, we form emulsions by condensing water vapor for 2 min onto oil-surfactant solutions of varying surfactant concentrations, and then measure their sizes after several months. Figure 4 shows the measured peak radius of nanoscale emulsions at 0 and 120 days. The emulsions are quite stable over this time period; the peak radius and polydispersity (especially for the emulsions containing the two highest surfactant concentrations) do not change considerably over the span of four months. The condensed droplets remain nanometric and do not grow substantially in size. The emulsion with the highest surfactant concentration (100 mM Span 80) remains the smallest in peak radius and in polydispersity after 120 days.

## Discussion

In summary, here we demonstrate a bottom-up assembly approach to creating stable nanoscale emulsions using condensation. By placing an oil bath in a humid environment and decreasing the temperature below the dew point, we induce water condensation on the oil surface; with sufficient surfactant, a nanoscale emulsion results. We observe experimentally that we are able to produce monodisperse, nanoscale water-in-oil emulsions by condensing water vapor onto dodecane containing a minimum concentration of 1 mM Span 80. The peak radius and polydispersity of the nanoscale emulsion may be controlled by varying the surfactant concentration and the time of condensation. The peak emulsion radius and the polydispersity both tend to decrease with an increase in surfactant concentration or a decrease in condensation time. In the present study, we systematically investigate Span 80. However, our method is not limited to this specific surfactant. For example, we performed similar condensation experiments with two additional oil-soluble surfactants: Span 85 and Brij 93. Both surfactants yielded nanoscale water-in-dodecane emulsions for surfactant concentrations well above the CMC (see Supplementary Figs. 5–6 for DLS measurements of Span 85 and Brij 93 emulsions). The resulting emulsions showed similar size ranges (peak radii around 100–150 nm, polydispersities less than 25%) to those obtained with Span 80.

We believe our emulsification approach is simple, scalable, and applicable to a wide range of materials systems. Our method provides control in tuning the dispersion size and polydispersity of nanoscale emulsions, while maintaining long-term stability. This technique may be further extended to formulate complex emulsions (e.g., w/o/w or o/w/o emulsions). Our approach may provide insights in the fields of colloids and self-assembly, with the potential to broadly impact the pharmaceutical, cosmetics, and processed foods industries.

## Methods

### Liquids used in the current study

The oil used in the current study was dodecane (*ρ*_o_ = 750 kg m^−3^, *n*_o_~1.42, *γ*_oa_~25.4 mN m^−1^, *μ*_o_~1.485 mPa s, *MP* = −10 °C). Here *ρ*_o_, *n*_o_, *γ*_oa_, *μ*_o_, *MP* denote density, refractive index, surface tension, dynamic viscosity, and melting point of the oil, respectively. The surfactant used in the study was Span 80 (mol. wt. = 428.6 g mol^−1^, *HLB = *4.3). All chemicals were purchased from Sigma-Aldrich.

### Interfacial tension measurements and CMC estimation

The interfacial tension between water and oil-surfactant solutions was measured using the pendant drop technique on Ramé-Hart goniometer (Model 500). Solutions were prepared by mixing oil and different concentrations of surfactants. The solutions were then transferred to quartz cuvettes for measurements. After injecting a water droplet in the cuvette through a hydrophilic needle, interfacial tension of water was recorded through the Ramé-Hart software. For a given surfactant concentration, surfactant adsorption at the oil/water interface resulted in steady decrease of interfacial tension to a final value after a finite adsorption time. This final value of interfacial tension was used to obtain the dependence of interfacial tension on surfactant concentration. The CMC of the surfactant was defined as the minimum surfactant concentration beyond which the interfacial tension value became relatively steady.

### Apparatus for performing condensation

A container with thermally conductive bottom plate (made of copper) and thermally insulating-hydrophobic rim (made of teflon) was fabricated for holding the liquids. The sample container was filled with 8 ml of oil-surfactant solution and then directly placed on a peltier cooler (TE Tech CP-061) within a humidifying chamber. The humidifying chamber was fitted with a humidity sensor (Sensirion SHT71) to monitor the temperature and humidity levels inside the chamber. The humidity chamber also served the function of preventing surface flow of the solution due to room air convection. The entire set-up was then placed under the microscope (Zeiss Axio Zoom.V16) for direct visualization of condensation phenomenon. A ‘Plan APO-Z 1.5’ lens along with an analyzer were used for obtaining images at magnification of ×260. The videos were recorded using Nikon D-800 camera in video recording mode at 1920 × 1080 size and 30 fps. For initiating condensation on the oil-surfactant solution, the peltier temperature was lowered below the room temperature (25 ± 1 °C) to a temperature of 2 ± 1 °C at a rate of ~5 °C/min. The humidity in the chamber was consistently measured at 75–80% signifying dew points of 13 ± 1 °C. The condensation time refers to the time the oil reservoir was maintained on the peltier, once the peltier temperature reached 2 ± 1 °C. The sample was immediately removed from the peltier after the water vapor had been condensed for the prescribed time (2, 10, or 30 min) to prevent any further condensation.

### Emulsion characterization

Optical microscopy was used to characterize the emulsion sizes for the cases where the droplet sizes were microscopic in size ranges. Videos were analyzed for droplet size estimation using ImageJ software. Emulsions with sizes below the optical detection limit were characterized using dynamic light scattering (DLS) at room temperature. DLS measurements were performed using DynaPro NanoStar, capable of identifying droplets in the size range of 0.2–2500 nm hydrodynamic radius. For a single sample, three different sample volumes were extracted. DLS measurements were acquired 10 times for each sample volume. From the DLS data, the droplet size and polydispersity of the solution were obtained.

### Data availability

All raw data for DLS measurements are provided as graphs in the Supplementary Information and may be provided in numerical form in a spreadsheet upon request.

## Electronic supplementary material

Supplementary Information

Description of Additional Supplementary Files

Supplementary Movie 1

Supplementary Movie 2
