# Supplementary material for: Creating nanoscale emulsions using condensation
Source: Nat Commun. 2017 Nov 8;8:1371. doi: 10.1038/s41467-017-01420-8 (PMC5678165; doi:10.1038/s41467-017-01420-8)
Supplement: Supplementary file 1 — Supplementary Information [file 41467_2017_1420_MOESM1_ESM.pdf]

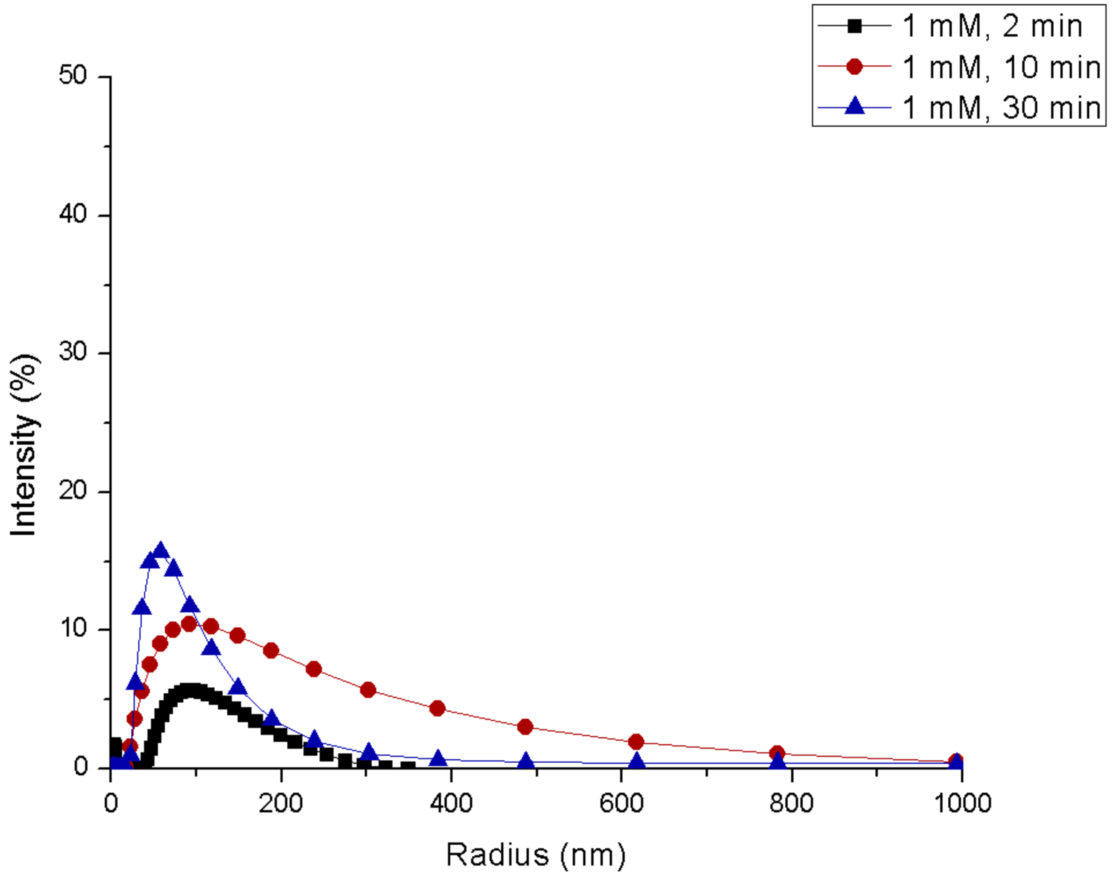

Supplementary Figure 1 | DLS measurements of emulsions formed by condensing water vapor onto dodecane containing 1 mM Span 80 for different condensation times. All experiments were performed at a relative humidity of 75-80% with the peltier cooler temperature set to 2 °C. The oil (8 mL) was kept in a copper bath with Teflon side-walls (to decrease the condensation rate on the side walls).

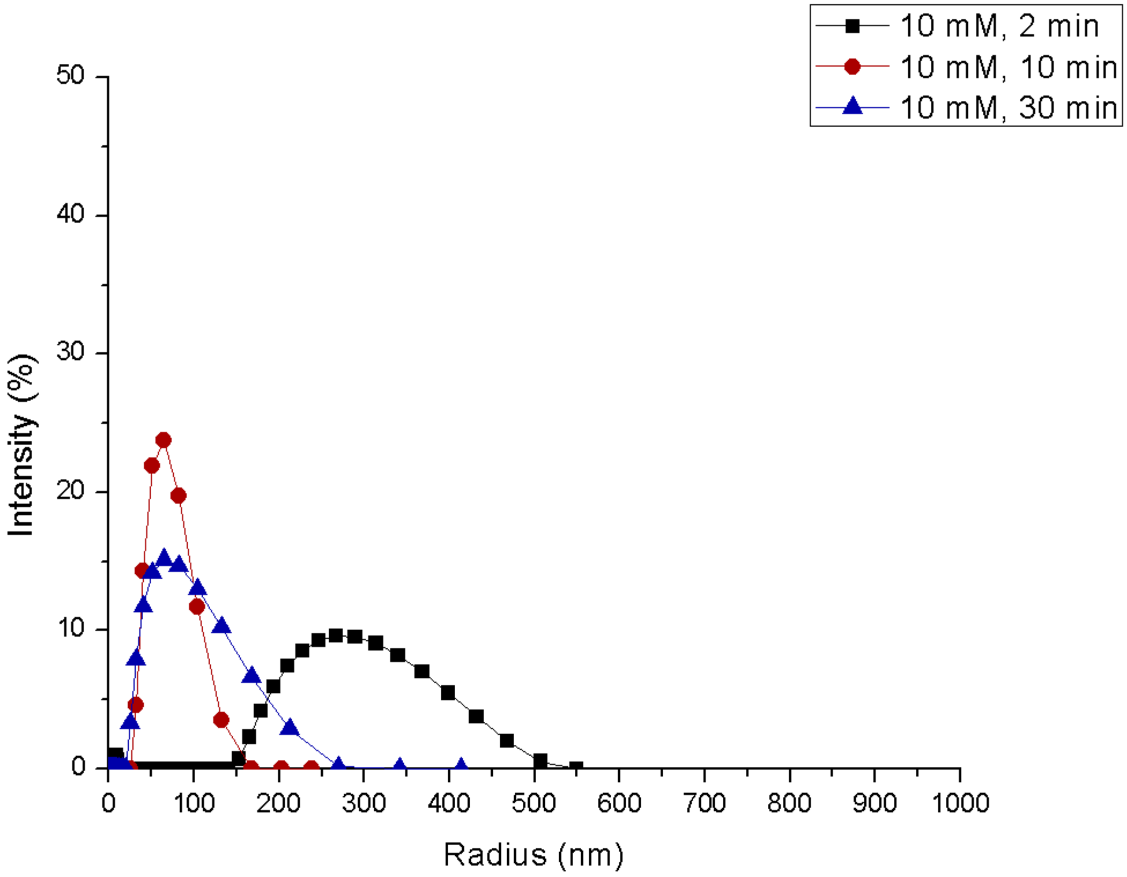

Supplementary Figure 2 | DLS measurements of emulsions formed by condensing water vapor onto dodecane containing 10 mM Span 80 for different condensation times.

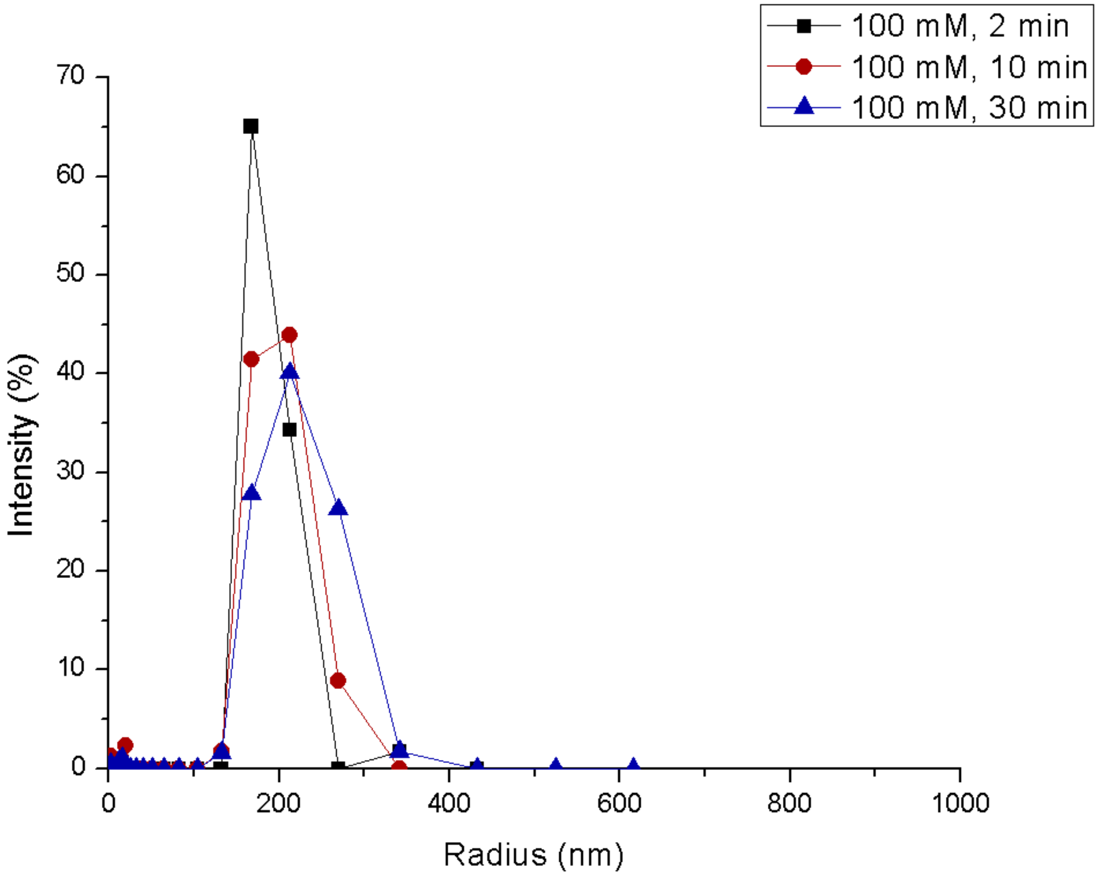

Supplementary Figure 3 | DLS measurements of emulsions formed by condensing water vapor onto dodecane containing 100 mM Span 80 for different condensation times**.**

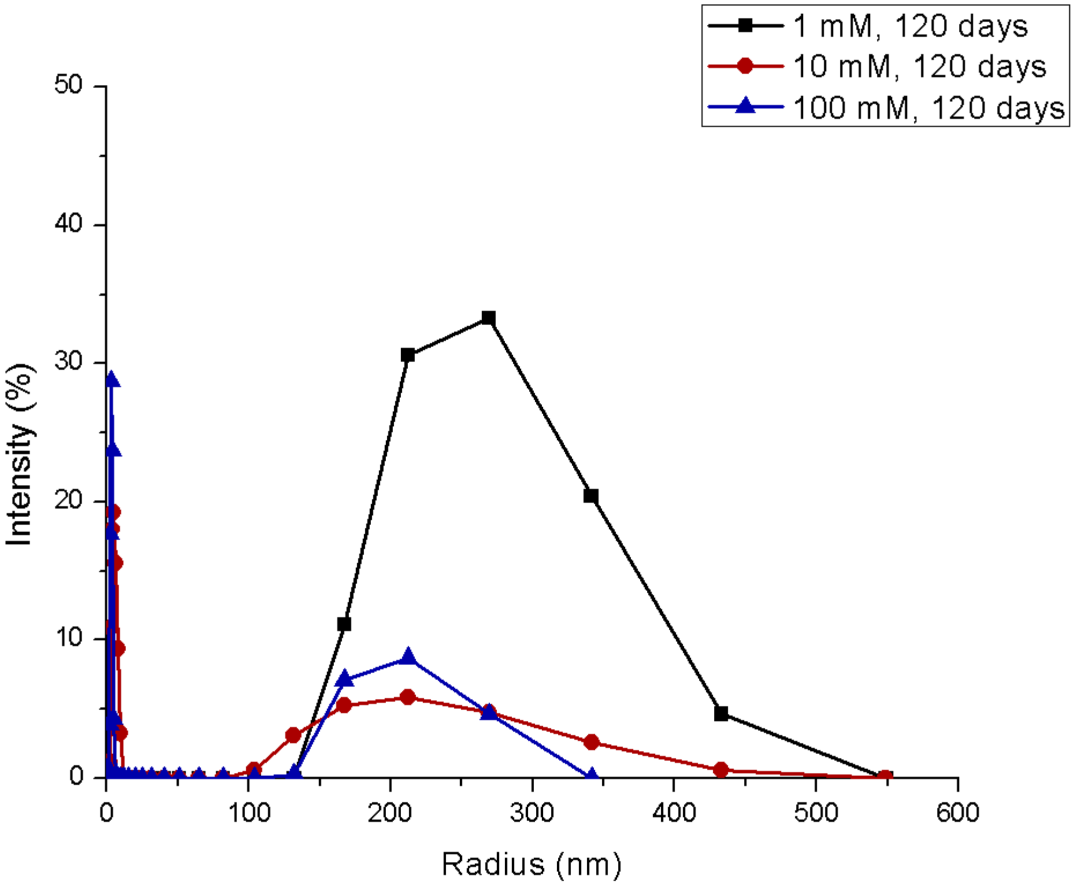

Supplementary Figure 4 | DLS measurements of emulsions formed 120 days previously by condensing water vapor onto dodecane containing 1 mM, 10 mM, and 100 mM Span 80 (condensation time: 2 min).

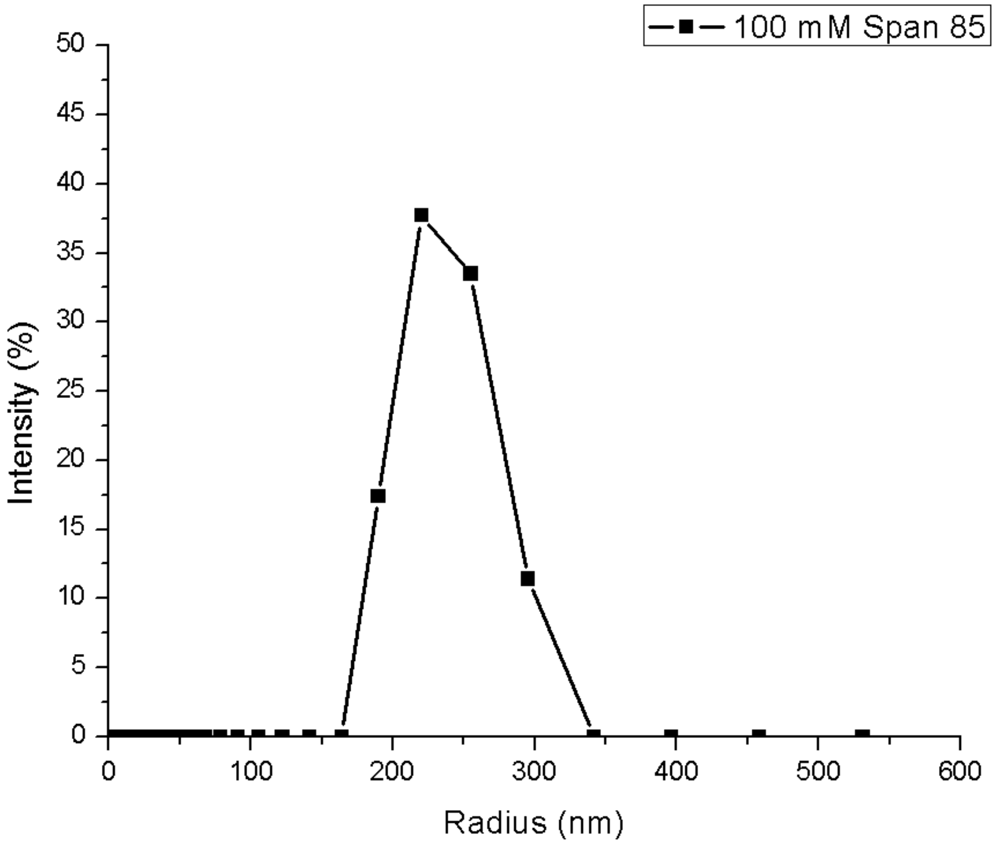

Supplementary Figure 5 | DLS measurement of emulsion formed by condensing water vapor onto dodecane containing 100 mM Span 85 (condensation time: 5 min).

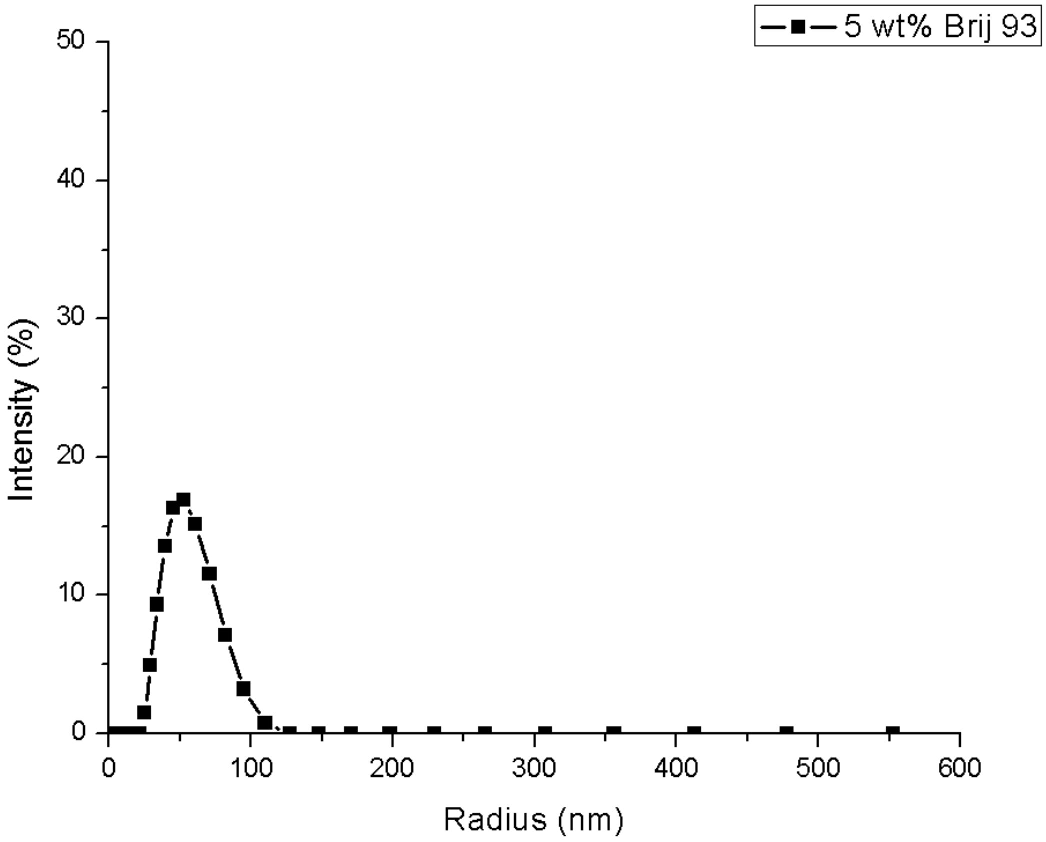

Supplementary Figure 6 | DLS measurement of emulsion formed by condensing water vapor onto dodecane containing 5 wt% Brij 93 (condensation time: 5 min)
