# Supplementary material for: Creating nanoscale emulsions using condensation
Source: Nat Commun. 2017 Nov 8;8:1371. doi: 10.1038/s41467-017-01420-8 (PMC5678165; doi:10.1038/s41467-017-01420-8)
Supplement: Supplementary file 2 — Description of Additional Supplementary Files [file 41467_2017_1420_MOESM2_ESM.pdf]

Description of Additional Supplementary Files

File Name: Supplementary Movie 1
Description: Condensation of Water Droplets onto Dodecane with and without Surfactant

File Name: Supplementary Movie 2
Description: Condensation of Water Droplets onto Dodecane with Different Surfactant Concentrations
